# The effect of daily calf stroking frequency during the postnatal period on the establishment of the human-calf relationship

**DOI:** 10.5713/ab.20.0745

**Published:** 2021-01-20

**Authors:** Satoko Wada, Michiru Fukasawa, Takashi Chiba, Tetsuro Shishido, Akitsu Tozawa, Shin-ichiro Ogura

**Affiliations:** 1 Graduate School of Agricultural Science, Tohoku University, Osaki, Miyagi, 989-6711, Japan; 2 Akita Prefecure, Akita, Akita, 010-8570, Japan; 3 Faculty of Life and Environmental Sciences, Teikyo University of Science, Adachi, Tokyo, 120-0045, Japan

**Keywords:** Animal Welfare, Calf, Human-calf Relationship, Postnatal Period, Stroking

## Abstract

**Objective:**

Stroking calves during the postnatal period could effectively improve human-calf relationships. The objective of this study is to examine how daily calf stroking frequency during the postnatal period affects the establishment of human-calf relationships.

**Methods:**

Six calves were stroked by a trainer for 6 minutes once daily for 5 days after birth (D1). Six calves were stroked by a trainer for 3 minutes twice daily for 5 days after birth (D2). A further four calves were stared at but not stroked as the control group. The overall stroking or staring duration was the same for all groups, at 6 min/d and 30 min over 5 days. The tests for reactions to the stationary trainer in an unfamiliar environment and avoidance distance measurements for an approaching trainer were conducted at 1 month and 3 months after the treatment.

**Results:**

Calves in both stroking groups approached significantly closer to the stationary trainer, vocalized less, and looked at the trainer shorter than the control group at 1 month. However, at 3 months, there was no significant difference between the D1 and the control group, whereas the D2 approached significantly closer to the trainer and vocalized less, and looked at the trainer for a shorter time than the control group. For the avoidance distance, the trainer could approach closer to both stroking groups than the control at 1 month, however, there was no difference among groups at 3 months.

**Conclusion:**

Our results suggested that the difference in the calf stroking procedure affected the established human-calf relationships, even though the total stroking duration was the same for all stroked calves. It is likely to be more effective to stroke more frequently than intensively when the aim is to establish better human-calf relationships within limited labor time.

## INTRODUCTION

The establishment of a good human-cattle relationship can have a substantial influence on the behavior, productivity, and welfare of cattle [1–3]. Hemsworth and Coleman [4] suggested that poor human-farm animal relationship would reduce the productivity in situations in which handling increase the animal’s fear for humans. Therefore, stockperson need to make cattle habituating to their presence and handling through repeated non-aversive contact [5] for establishing good relationships. However, there is limited time available to repeatedly contact them because of the decrease in time spent per cattle due to the increase in the number of cattle per stockperson [1]. Therefore, how to establish better relationships with each cattle within limited labor time is one of the challenging issues in modern farm management.

Previous studies showed gentle contact, such as stroking calf and speaking with a gentle voice, could reduce the fear of cattle for humans and improve human-cattle relationships [6,7]. Gentle contact during sensitive periods, when animals are susceptible to particular experience [8,9], is effective in reducing the fear of humans and calming animals [10,11]. There are reportedly three sensitive periods for habituating cattle to handling: postnatal period [11,12], at weaning [11,13,14], and first calving [15]. Previous studies already showed that stroking calf with speaking gentle voice during the postnatal period could effectively improve human-calf relationships [12,16–18], therefore, stroking during the postnatal period could be an effective solution for establishing better relationships with each calf within a limited time.

However, the stroking procedure related to total stroking duration, such as stroking duration per trial, daily trial frequency, and the number of days, varied among previous studies. For example, Probst et al [17] stroked each calf for 10 min twice a day for 3 consecutive days after birth, while Lürzel et al [18] stroked each calf for 3 min once a day for 14 consecutive days after birth. However, the effects of variation in stroking procedure on the establishment of human-calf relationships have not been discussed. In learning theory, it is well-known that variation in learning procedure strongly influences results [19,20]. By clarifying the effect of each factor relating to total stroking duration, we would show the optimum stroking procedure to maximize the efficiency of establishing human-calf relationships even in limited labor time.

Therefore, the objective of this study is to examine how daily calf stroking frequency during the postnatal period affects the establishment of human-calf relationships when both groups experienced the same total stroking duration. We will also discuss time-efficient and effective stroking procedure for establishing better human-calf relationships.

## MATERIALS AND METHODS

The protocol of this study was approved by Tohoku University Animal Experiment Committee (2017AgA-036).

### Animals and management

Sixteen calves (8 Japanese Black and 8 Japanese Shorthorn) were subjects of this experiment. They were born between July and November 2017. The experiment was carried out at Kawatabi Field Center, Graduate School of Agricultural Science, Tohoku University. Calves were born in calving pen A (4 m×4 m) or B (4 m×8 m). Fourteen calves were born naturally and sucked colostrum voluntarily from their dam. One calf was assisted to suck colostrum by a stockperson. A stockperson assisted the birth of the remaining calf and fed it 225 g of powdered colostrum. After birth, each dam-calf pair was kept in the same calving pen (A or B), then moved into the group pen (14 m×8 m) when the calf was about 20 days old. In the group pen, eight dam-calf pairs were kept, of which 2 to 6 pairs were kept for this experiment. Dams were fed hay and concentrated feed. No special feed was given to the calves.

### Stroking treatment

This experiment consisted of two phases. One was the postnatal stroking treatment phase, and another was the assessment test phase at 1 month and 3 months after the treatment phase.

In this study, two stroking treatment groups, which were once-a-day (D1) or twice-a-day (D2), and a control group were employed. Both stroking treatments were carried out for 5 consecutive days from the day after birth by the same female trainer (20’s) who was not a stockperson. Each group contained 50% Japanese Black and 50% Japanese Shorthorn calves. In the D1, 6 calves received 6 min stroking once a day at either 10:00 or 17:00. The stroking time was consistent within each calf. In the D2, 6 calves received 3 min stroking twice a day (10:00 and 17:00). The overall stroking duration was the same for D1 and D2, at 6 min/d, and 30 min over 5 days. In both stroking groups, the trainer stroked the calf across its back (40%), belly (30%), and neck (30%) while talking to it gently [11]. When calves avoided being stroked, the trainer left the calf for a short time and processed stroking again. During stroking, the dam was tethered or kept away from the trainer by an assistant for safety reasons. The dam could not approach the calf but could see and hear it. In the control group, 4 calves were stared at by the trainer from 3 m away for 6 min once a day (10:00 or 17:00). The reason for carrying out this treatment is to control for the effect of the trainer’s presence on the calves. This treatment was also carried out for 5 consecutive days from the day after birth. The overall staring duration was at 6 min/d and at 30 min over 5 days which was as same as the overall stroking duration.

Calves received the minimum possible contact with stockpersons during the whole experimental period: feeding twice a day (9:00 and 16:00); measurement of body weight once a month; disease treatment. Twelve calves (D1, 4; D2, 5; control, 3) had symptoms of diarrhea during the experimental period and were treated with intramuscular antibiotic injections and intravenous hepatic stimulant injections. Also, one calf that contracted umbilical corditis within a week after birth was treated with an intramuscular antibiotic injection.

### Assessment tests

The tests for reactions to a stationary trainer in an unfamiliar environment and the avoidance distance measurements for an approaching trainer were conducted at 1 month and 3 months after the treatment. Both tests were used to assess the human-animal relationship [21].

#### Reactions to the stationary trainer

**Figure 1:**
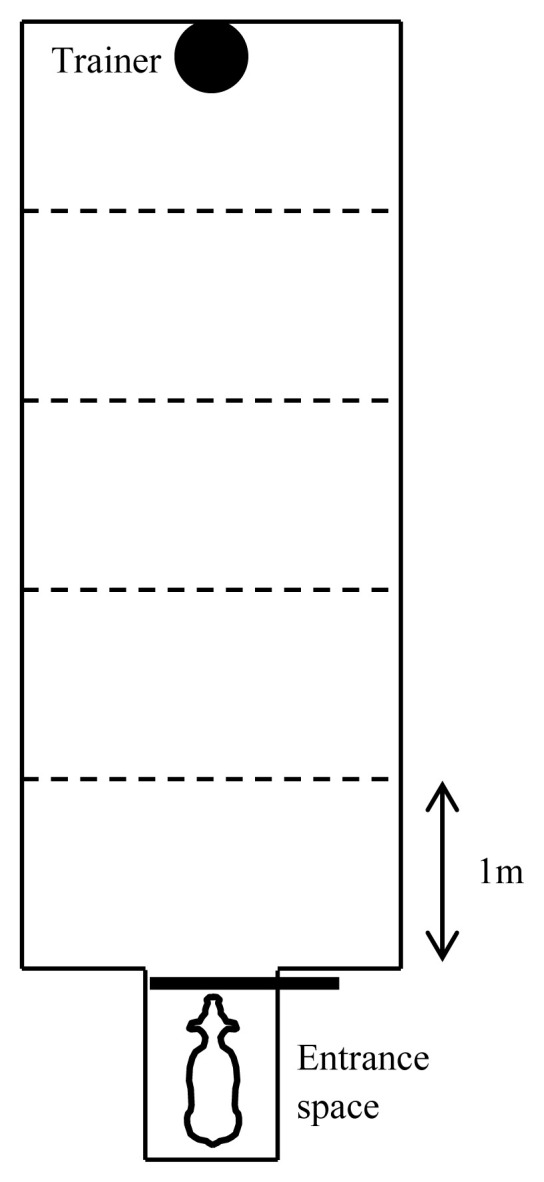
Diagram of the arena for the test for reactions to the stationary trainer (width, 200 cm; length, 500 cm; height, 180 cm). Solid and bold lines represent the wood panel and slide gate of the calf entrance, respectively. The trainer stood at the position of the black dot during a test.

In the test for reactions to a stationary trainer, we measured the behavioral reactions of the calf to the stationary trainer under the unfamiliar environment. A novel arena (Figure 1) was constructed with wooden panel walls and concrete floors. The calf entrance space was attached to the short side of the arena. The trainer stood on the side opposite the entrance space. Calf behavior during the test was recorded by two cameras (WTH-HR872S, WIRELESS Tsukamoto Co. Ltd, Tsu, Japan) and a recorder (WTM-DH 620-2TB, WIRELESS Tsukamoto Co. Ltd, Japan).

Before starting the test, the calf was left alone in the entrance space for a minute to calm them down. Then the slide gate of the entrance space was opened to allow the calf into the test arena. The test started when the calf’s forefoot first entered the arena and lasted for 5 min. When the calf did not voluntarily enter the arena within 1 min after opening the gate, the assistant pushed their back to drive it into the arena.

The following behaviors were observed during the test:

- The closest approach distance to the trainer (cm): the shortest distance measured in 50 cm increments between the calf’s nearest forefoot and trainer’s foot during the test.
- The number of vocalizations during the test (times)
- The duration of looking at the trainer (seconds): the duration when the calf was standing still and looking at the trainer with its head up
- The number (times) and duration (seconds) of exploration: the number and duration of sniffing floors, walls, and trainer in the arena
- The number of excretions (times): the number of defection and urination

#### Avoidance distance

Avoidance distance was measured by the trainer in the group pen. At first, the trainer entered the pen and stayed for 1 min to allow the standing calf to see the trainer. The trainer then approached the calf from the front and 5 m away at a speed of 1 step/s. Both arms were extended in front of the trainer at an angle of about 45°, with the back of the hand pointing forwards [3]. When the calf withdrew, the distance between the calf’s nearest forefoot and trainer’s foot was measured in 10 cm increments as the avoidance distance. If the calf did not withdraw and accept the contact, the measurement was finished, and an avoidance distance of 0 cm was assigned. Avoidance distance was measured three times within a day for each calf. To avoid any influence of the previous measurement, it took more than 3 minutes between the measurements. The average of 3 measurements was used as a representative value of each calf for statistical analysis.

### Statistical analysis

Data were analyzed using IBM SPSS Statistics software (version 23). The normality of data distribution was tested using Shapiro-Wilk methods. No measurement was normally distributed except exploring duration. We used a generalized linear model including the fixed effects of the breed, treatment, and month, and the interactions between treatment and month. We assumed a normal distribution for the duration of exploring, and Poisson distribution with a log link function for the other measurements. We conducted post-hoc testing with the least significant difference method when a significant effect was observed.

## RESULTS

Some calves were hard to stroke on the first day, however all calves accepted the stroking on the last day. During the stroking treatment phase, the behavior of the calves towards the trainer did not differ between treatments.

**Figure 2:**
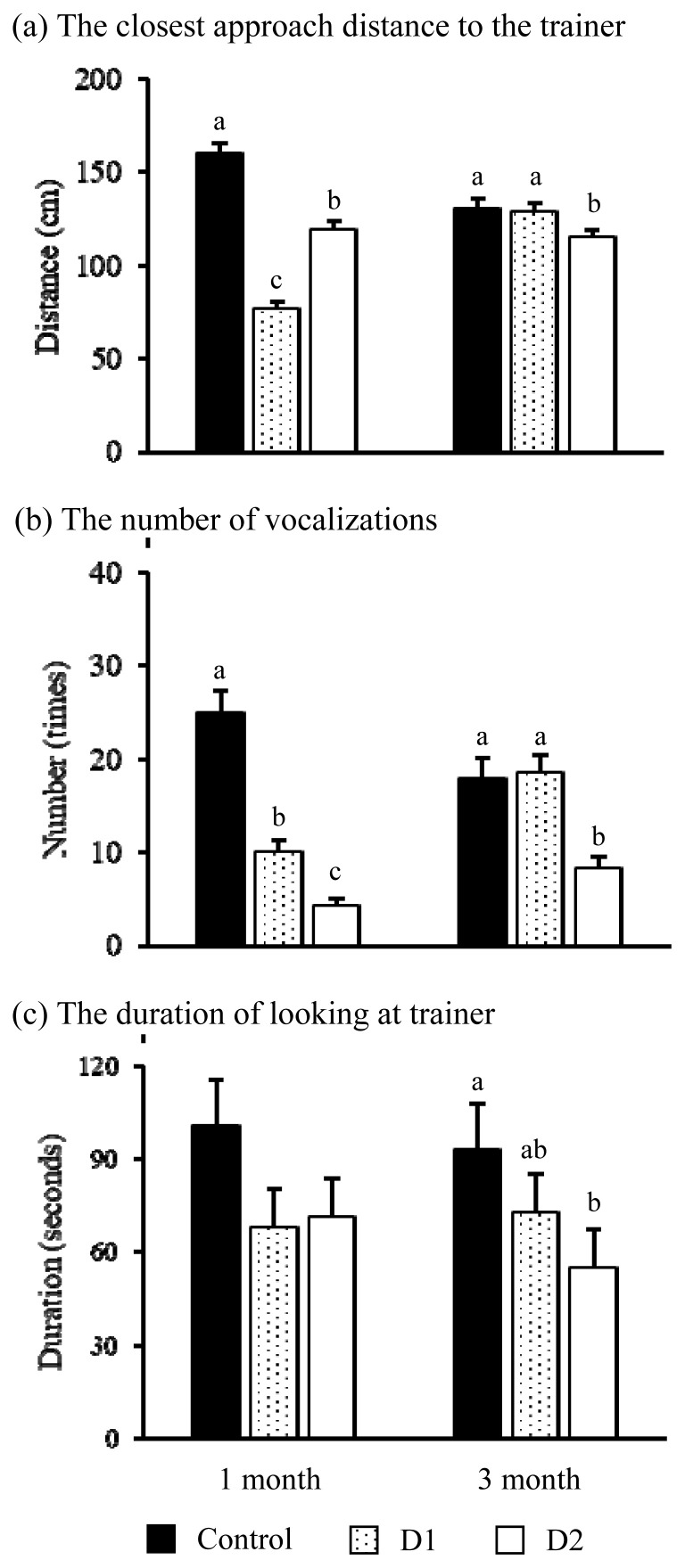
The comparison of (a) the closest approach distance to the trainer, (b) the number of vocalizations, and (c) the duration of looking at the trainer among the control calves (control, closed bar), calves stroked once a day (D1, dotted bar), and calves stroked twice a day (D2, open bar) in each test month in the test for reactions to the stationary trainer. ^a–c^ Represents a significant difference among treatments within the month (p<0.05).

The results of the test for reactions to the stationary trainer are shown in Figure 2. There was a significant interaction between treatment and month on the closest approach distance (p<0.001, Figure 2a), the number of vocalizations (p< 0.001, Figure 2b), and the duration of looking at the trainer (p<0.01, Figure 2c), respectively. However, there was no significant interaction on the number and duration of exploration, and the number of excretions.

At 1 month, both stroking groups approached significantly closer to the trainer (p<0.001 for both groups), vocalized less (p<0.001 for both groups), and calves in D1 tended to look at the trainer for a shorter time (p = 0.09) than the control group. Furthermore, the D1 significantly approached closer to the trainer (p<0.001) and vocalized more (p<0.001) than the D2.

However, at 3 months, the D2 approached significantly closer to the trainer (p<0.01) and vocalized less (p<0.01), and looked at the trainer for a shorter time (p<0.05) than the control group.

**Figure 3:**
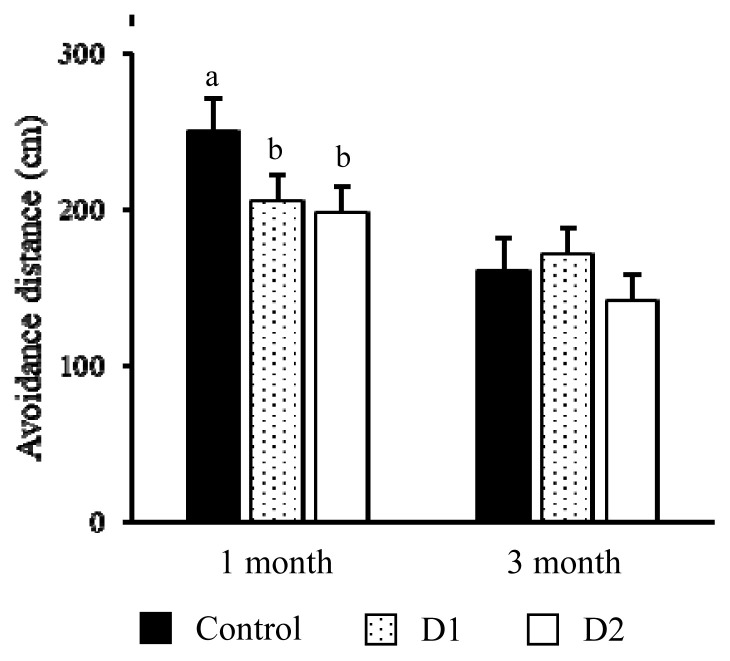
The comparison of the avoidance distance to the approaching trainer among the control calves (control, closed bar), calves stroked once a day (D1, dotted bar), and calves stroked twice a day (D2, open bar) in each test month. ^a,b^ Represents a significant difference among treatments within the month (p<0.05).

For the avoidance distance in group pen (Figure 3), there was a significant interaction between treatment and month (p<0.001). At 1 month, the avoidance distances for both stroking groups were shorter than the control group (p<0.01 for both groups). At 3 months, there was no significant difference between both stroking groups and the control group.

## DISCUSSION

Our results suggested that a short but frequent stroking procedure would be more effective than intensive stroking for establishing persistent and good human-calf relationships within the same stroking duration. The fear of humans at 1 month can be alleviated by stroking calf treatment during the postnatal period in both treatment groups. Calves in both treatment groups showed that voluntarily approached closer to the trainer and vocalized less in the test for reaction to the stationary trainer, and shorter avoidance distance than the control group. The closest approach distance to stationary human in the novel arena represents a better human-cattle relationship [1,21,22]. Kosako and Imura [12] indicated that vocalizations represented anxiety and fear caused by isolation from the herd. Coulon et al [10] reported that isolated sheep vocalized less in the test arena when they were with a familiar caregiver, compared to alone, because a familiar caregiver could serve as an effective substitute for the pen mate. Avoidance distance also represents a calf’s fear of humans [22], and previous studies also reported that stroking treatment during the postanal period reduces the calf’s fear of humans and shortened the avoidance distance [2,3,17]. However, calves only in the D2 showed that voluntarily approached closer to the trainer, vocalized less, and looked at the trainer for a shorter time than the control calves until 3 months after stroking under test situation. Waiblinger et al [23] reported that cattle look at the subject longer when they feel fear or a need for caution. Despite statistical significance, however, there were little differences in the closest approach distances between D2 and others in 3 months, and seems to be no biological significance. The reason for this significance might be that the closest distance was measured in 50 cm increments, which might cause inadequate data structure. Therefore, the result of the closest distance should be re-examined with finer interval measurements.

One of the reasons why the effect was different between stroking frequencies even though total stroking duration was the same would be the chance and opportunity to learn that humans are not aversive. Cattle could learn and habituate to human presence as non-aversive stimulus through repeated gentle contact and talking [5]. Kosako and Imura [16] found that exploration behavior of calves was increased for the 2 or 3 days after birth; short but frequent stroking would provide more opportunities to explore and learn that humans are non-aversive. Additionally, Smolen et al [19] showed that frequent training leads to more robust memory formation than intensive training in rats, even where total training duration was the same. Similarly, also in rats, frequent training procedures promoted exploration behavior for the learning of objects compared to the intensive procedure [20].

## CONCLUSION

The stroking calves during the postnatal period could be useful in establishing a better human-calf relationship. This study suggested that differences in the duration and frequency of stroking procedure affected the established human-calf relationships, although total stroking duration was the same for all stroked calves. Calves stroked in short but frequent bout could establish persistent better relationships after 3 months had gone by. In contrast, calves stroked intensively also could establish better relationships, however, this had disappeared by 3 months. Our results implicated that it is likely to be more effective to stroke more frequently than intensively for establishing a persistent better human-calf relationship within limited a labor time.
